# Laminin-111-derived peptide conjugated fibrin hydrogel restores salivary gland function

**DOI:** 10.1371/journal.pone.0187069

**Published:** 2017-11-02

**Authors:** Kihoon Nam, Christina L. Maruyama, Ching-Shuen Wang, Bryan G. Trump, Pedro Lei, Stelios T. Andreadis, Olga J. Baker

**Affiliations:** 1 School of Dentistry, University of Utah, Salt Lake City, Utah, United States of America; 2 Department of Chemical and Biological Engineering, University at Buffalo, The State University of New York, Buffalo, New York, United States of America; 3 Department of Biomedical Engineering, School of Engineering and Applied Sciences, University at Buffalo, The State University of New York, Buffalo, New York, United States of America; 4 Center of Bioinformatics and Life Sciences, University at Buffalo, The State University of New York, Buffalo, New York, United States of America; University of South Carolina, UNITED STATES

## Abstract

Hyposalivation reduces the patient quality of life, as saliva is important for maintaining oral health. Current treatments for hyposalivation are limited to medications such as the muscarinic receptor agonists, pilocarpine and cevimeline. However, these therapies only provide temporary relief. Therefore, alternative therapies are essential to restore salivary gland function. An option is to use bioengineered scaffolds to promote functional salivary gland regeneration. Previous studies demonstrated that the laminin-111 protein is critical for intact salivary gland cell cluster formation and organization. However, laminin-111 protein as a whole is not suitable for clinical applications as some protein domains may contribute to unwanted side effects such as degradation, tumorigenesis and immune responses. Conversely, the use of synthetic laminin-111 peptides makes it possible to minimize the immune reactivity or pathogen transfer. In addition, it is relatively simple and inexpensive as compared to animal-derived proteins. Therefore, the goal of this study was to demonstrate whether a 20 day treatment with laminin-111-derived peptide conjugated fibrin hydrogel promotes tissue regeneration in submandibular glands of a wound healing mouse model. In this study, laminin-111-derived peptide conjugated fibrin hydrogel significantly accelerated formation of salivary gland tissue. The regenerated gland tissues displayed not only structural but also functional restoration.

## Introduction

Hyposalivation is the condition of having insufficient or reduced saliva production [1]. There are several causes of hyposalivation, including the use of medication, radiation therapy for head and neck cancer treatment and autoimmune disorder (*e*.*g*. Sjögren's syndrome) [2]. Salivary gland damage caused by blunt injury, chemotherapy and radiotherapy leads to hyposalivation thereby reducing the quality of life of many patients, as saliva is important for maintaining oral health [3, 4]. Current treatments for hyposalivation are limited to medications such as the muscarinic receptor agonists, pilocarpine, and cevimeline [5]. However, these therapies only provide temporary relief. Therefore, alternative therapies are essential to restore salivary gland function. Tissue damage, as a result of injury or disease, is a major health problem that can lead to irreversible organ failure [6, 7]. A possible treatment to completely restore tissue function is the use of bioengineering approaches [8]. The use of natural polymeric hydrogels are promising scaffolds for tissue engineering because of their biocompatibility, biodegradability and biological functions. Fibrin is the major extracellular matrix protein involved in blood clotting. Fibrin hydrogel (FH) can be easily made by mixing fibrinogen with thrombin at 37°C. The mechanical properties of FH can be controlled by changing the concentration of fibrinogen [9] and can be decorated with bioactive signals making it an ideal scaffold for tissue engineering and regenerative medicine [10–13]. Moreover, fibrinogen and thrombin can be harvested from the patient's own blood, therefore FH can eliminate the immune rejection and viral transmission [14]. Additionally, FH can be used for drug delivery, cell therapy, and gene therapy to the damaged tissues [15–18].

Previous studies have shown that laminin proteins from the basement membrane play a key role in embryonic epithelium development [19]. Particularly, laminin-111 (L1) is significantly upregulated during embryogenesis in a variety of tissues to allow for cell attachment and promote tissue remodelling [20]. However, L1 expression is downregulated shortly after birth and becomes limited to few tissues such as the brain and kidney. Our previous studies demonstrated that that highly purified L1 improved growth, organization, and differentiation of salivary cell clusters grown *in vitro* [21]. However, L1 is not suitable for clinical applications as some protein domains are known to promote immunogenic response that may outweigh the potential benefits provided by the whole molecule [22, 23]. Therefore, we used several L1 peptides to evaluate their ability to promote formation of three-dimensional salivary cell clusters using FH as scaffold *in vitro*. Particularly, two L1 peptides, YIGSR and A99 demonstrated improved lumen formation and increased cell attachment, respectively [21]. Furthermore, when using a combination of these peptides *in vivo*, a damaged mouse submandibular gland (mSMG) was able to grow and partially differentiate after 8 days. Despite these encouraging findings, however, several key indicators of intact salivary gland functioning were not noted, which was thought to be attributable to the limited duration of the observation period [24]. In response to these concerns, the current study expands on our previous work by extending the duration of the prior experiments (*i*.*e*., from 8 days to 20 days), thereby allowing for saliva secretion to occur and for acinar functional markers (*i*.*e*., AQP5 and TMEM16) as well as indicators of blood vessel and nerve formation (PECAM-1 and β-tubulin III, respectively) to be detected.

## Materials and methods

### Materials

Lyophilized fibrinogen from human plasma and Millex syringe filter (0.22 μm) were purchased from EMD Millipore (Billerica, MA). Lyophilized thrombin from bovine plasma, calcium chloride, ε-aminocaproic acid (εACA), and Alcian Blue 8GX were purchased from Sigma-Aldrich (St. Louis, MO). Peptides were synthesized by University of Utah DNA/Peptide synthesis core facility. Spectra/Por 7 dialysis membrane (MWCO = 3.5 kDa) was purchased from Spectrum Laboratories (Rancho Dominguez, CA). Coomassie Brilliant Blue R-250 was purchased from Genlantis (San Diego, CA). Sulfosuccinimidyl 6-(3'-(2-pyridyldithio)propionamido)hexanoate (Sulfo-LC-SPDP) and DyLight 680 NHS-ester were purchased from Thermo Fisher Scientific (Newington, NH). Mini-PROTEAN TGX precast electrophoresis gel was purchased from Bio-Rad (Hercules, CA). TO-PRO-3 iodide, Alexa Fluor 488 conjugated anti-rabbit IgG secondary antibody and Alexa Fluor 568 conjugated anti-mouse IgG secondary antibody were purchased from Invitrogen (Carlsband, CA). Rabbit anti-aquaporin 5 (AQP5), rabbit anti-TMEM-16A, rabbit anti-PECAM-1, mouse anti-cytokeratin 7, mouse anti- β-tubulin III, rabbit anti-Ki67 and Picrosirius Red Stain Kit were purchased from Abcam (Cambridge, MA). Mouse Na^+^/K^+^-ATPase antibody was purchased Santa Cruz Biotechnology (Santa Cruz, CA).

### Synthesis of peptide or DyLight 680 conjugated fibrinogen

Peptide and DyLight 680 conjugated fibrinogen were prepared as previously described [21, 24]. Briefly, two L1 derived peptides (A99: CGGALRGDN-amide, YIGSR: CGGADPGYIGSRGAA-amide) were synthesized on a peptide synthesizer using Fmoc solid-phase peptide. In order to create a thiol-reactive fibrinogen, the primary amine groups were activated with 7.2 equivalents of sulfo-LC-SPDP for 1 h at room temperature. Then, the excess cross linker and its by-products were removed by dialysis (molecular weight cut-off (M.W.C.O) = 3.5 kDa). For peptide conjugation, LC-SPDP activated fibrinogen was reacted with 2 equivalents of peptide for 18 h at room temperature. Finally, peptide conjugated fibrinogen was dialyzed against ultrapure water (M.W.C.O. = 3.5 kDa) and filtered using a 0.22 μm syringe filter. Then, the product was lyophilized. To monitor fibrin hydrogels stability, fluorescently conjugated fibrinogen was also produced. Lyophilized fibrinogen was reacted with DyLight 680 for 1 h at room temperature. Non-reacted reagent was removed by dialysis (M.W.C.O = 3.5 kDa). Then, the product was filtered using a 0.22 μm syringe filter and lyophilized. All fibrinogens were stored at −80°C until use. The reactions were monitored and confirmed using thin-layer chromatography, UV-Vis spectrum and static light scattering data, as described previously [21]. Based on these data, six peptides and five DyLight 680 were conjugated to a fibrinogen molecule. In addition, the final percent yields for YIGSR conjugated fibrinogen, A99 conjugated fibrinogen, and DyLight 680 conjugated fibrinogen were 83.32%, 86.73%, and 78.15%, respectively.

### Hydrogel preparation

Laminin-111-derived peptides conjugated fibrin hydrogel (L_1p_-FH) was fabricated by mixing YIGSR-conjugated fibrinogen (1 mg/mL) and A99-conjugated fibrinogen (1 mg/mL), DyLight 680 conjugated fibrinogen (0.5 mg/mL) and plasma-derived bovine thrombin (2.5 U/mL) in TBS with CaCl_2_ (2.5 mM) and εACA (2 mg/mL). For the FH treated group, fibrinogen (2 mg/mL), DyLight 680 conjugated fibrinogen (0.5 mg/mL) and plasma-derived bovine thrombin (2.5 U/mL) in TBS with CaCl_2_ (2.5 mM) and εACA (2 mg/mL) were used.

### Rheological parameters

The rheological properties are highly dependent on the macromolecular structure, which in turn affects the mechanical of hydrogels [25]. To this end, rheological measurements were performed on a stress-controlled AR 2000ex rheometer (TA Instruments, Crawley, UK) using a stainless-steel cone and plate geometry (4° cone angle; 20 mm cone diameter and truncation height of 114 μm) at 37°C with a continuous strain value of 0 to 100. One hundred microliter of mixture was applied to the bottom plate, and a solvent trap filled with water was used to prevent sample evaporation. Then, the modulus of elasticity (G’) and the strain (%) were recorded for 5 min.

### Animals

Female C57BL/6 mice 5-7-weeks-old, weighing 17–22 g, were purchased from the Jackson Laboratory (Bar Harbor, ME). Then, 28 mice were randomly distributed into 4 groups: untreated, FH treated, L_1p_-FH treated and sham surgery control. Animals were housed in cages in a room with a controlled environment (12-hour day/night cycles) and provided with a standard pellet diet and water. All animal management, anesthesia, and surgeries followed the protocol (protocol number: 17–05001) approved by the Institutional Animal Care and Use Committee (IACUC) at the University of Utah. Carprofen (5 mg/kg/day) was used to manage incision-induced pain after surgery.

### Surgical procedure and scaffold stability

**Fig 1:**
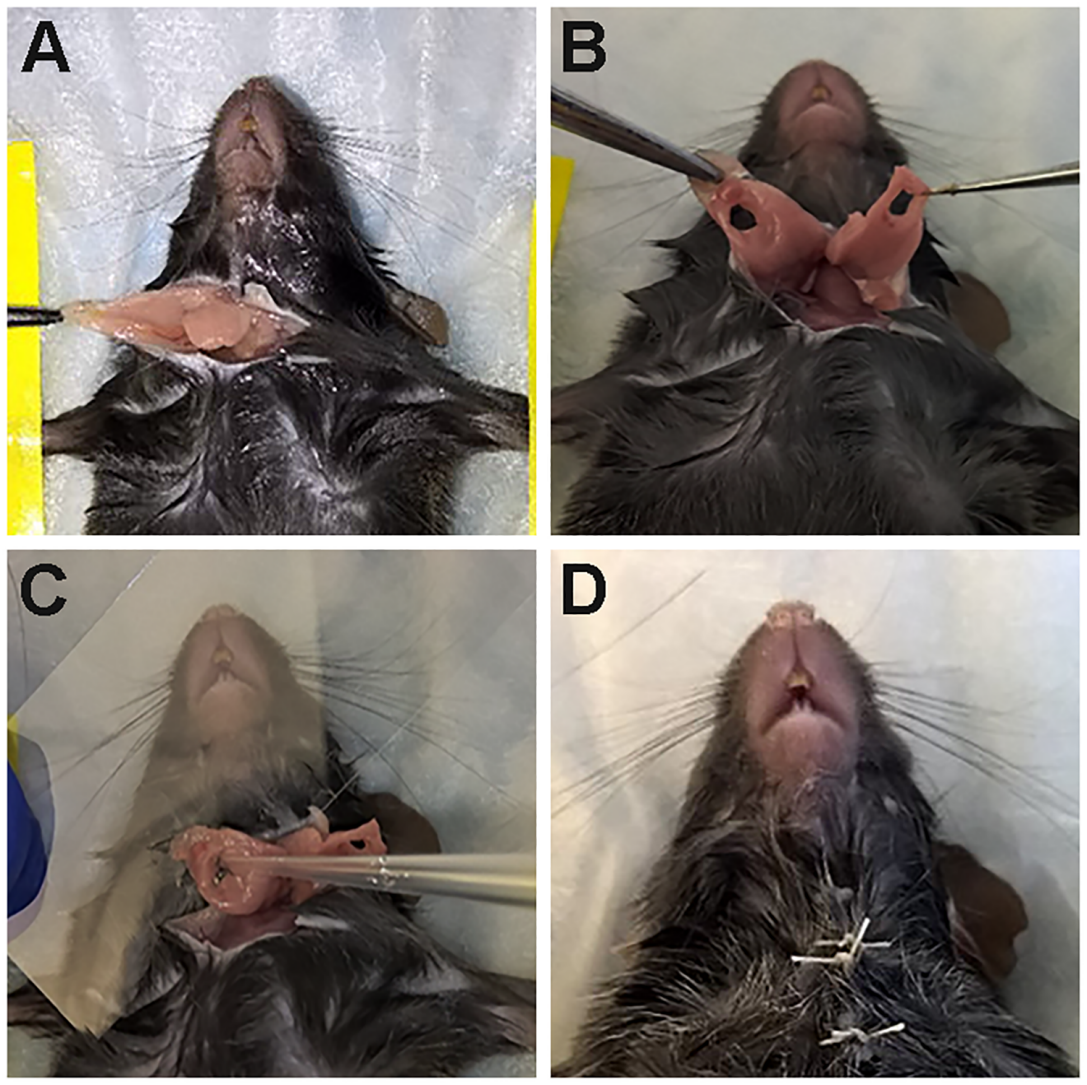
Procedure to create wounded SMG model. (A) A skin incision of approximately 1 cm in length was made along the anterior surface of the neck, mSMG were exposed, (B) a 3-mm diameter biopsy punch was performed, surgical wounds were completed, (C) wounds were filled with or without L_1p_-FH or FH and (D) the skin incision was sutured.

C57BL/6 mice were anesthetized with 3% isoflurane with an oxygen flow rate set at 2.0 L/min. Then, a skin incision of approximately 1 cm in length was made along the anterior surface of the neck (Fig 1A). Subsequently, mSMGs were exposed and the surgical wounds were created using a 3-mm diameter biopsy punch (Fig 1B). To determine the effects of FH, 20 μL of this scaffold was added at the surgical wounds where a coverslip was placed underneath to prevent leakage (Fig 1C). The different scaffolds used in the experiments are listed in Table 1. Finally, the skin incision was sutured and post-surgical studies at day 20 were performed (Fig 1D).

**Table 1:** Composition of fibrin hydrogels.

Hydrogels	Composition (picomole)			
	Fibrinogen	DyLight 680	YIGSR	A99
FH	146.3	144.9	-	-
L_1p_-FH	143.6	144.9	342.6	345.5

Twenty microliter of mixture was added at the surgical wounds.

To monitor scaffold stability *in vivo*, FH were conjugated with Dylight 680. Fluorescent intensity of the scaffold was monitored in a Xenogen IVIS 100 Bioluminescent Imager (University of Utah, Center for Quantitative Cancer Imaging) at post-surgery day 1, 3, 8 and 20. Fluorescent images were acquired with a filter set using excitation/emission at 692/712 nm.

### Measurement of body weight

In order to monitor post-surgery body mass, mice were weighed at the start of each experiment and data was collected for 20 days. Statistical significance was assessed by two-way ANOVA (*p* < 0.01) and Dunnett's post-hoc test for multiple comparisons to the untreated group.

### Salivary secretion rate

Mice were anesthetized with ketamine (100 mg/kg) and xylazine (5 mg/kg), and injected with pilocarpine (10 mg/kg) via intraperitoneal injection to stimulate saliva secretion. Then, whole saliva was collected and measured using a 200 μl pipette. Statistical significance was assessed by one-way ANOVA (*p* < 0.01) and Dunnett's post-hoc test for multiple comparisons to the untreated group.

### Saliva protein composition

To determine the saliva composition of each condition, 15 μg of saliva protein from each group were fractionated by SDS-PAGE. Saliva samples were denatured at 95°C for 5 min in a sample loading buffer. The denatured samples were loaded onto the Mini-PROTEAN TGX precast electrophoresis gel (Any kD^™^, Bio-Rad) and subjected to electrophoresis in 25 mM Tris/192 mM Glycine buffer with 0.1% SDS (w/v) at 100 V for 70 min. The electrophoresis gel was fixed in a solution of 25% ethanol, 15% formaldehyde, 60% water for 1 h and re-fixed with 50% methanol, 40% water, 10% glacial acetic acid for overnight. For general protein staining, the gel was stained with 0.25% Coomassie Brilliant Blue R-250 in 50% (v/v) methanol, 10% (v/v) glacial acetic acid for 1 h and destained overnight in 20% (v/v) methanol and 10% (v/v) acetic acid. For mucin staining, the fixed gel was stained with 0.5% Alcian Blue 8GX in 2% (v/v) acetic acid for 1 h. Then, the gel was destained overnight in 20% (v/v) methanol and 10% (v/v) acetic acid. Protein images of gels were captured using a Chemi Doc^mp^ imaging system (Bio-Rad). ImageJ was used to perform the image analysis. All statistical analyses were performed with GraphPad Prism 6.

### Histological studies

mSMG tissues were immersed in 10% formalin at room temperature for one day, dehydrated in serial ethanol solutions (50%, 70% and 100% for 2 h each), embedded in paraffin wax and cut into 7 μm sections. mSMG sections from each group were deparaffinized with xylene and rehydrated with serial ethanol solutions (100%, 70% and 50%) and distilled water. Then, hematoxylin and eosin (H&E) and picrosirius red staining were performed, and tissue sections were subjected to a blind histopathological analysis using a Leica DMI6000B inverted microscope (Leica Microsystems, Wetzlar, Germany) as well as an Olympus BX53 Light Microscope (Olympus America, Center Valley, PA). In addition, the ratio of acinar structures to ductal structures was analyzed using ImageJ and GraphPad Prism 6.

### Confocal microscopy

Deparaffinized sections were incubated in sodium citrate buffer (10 mM sodium citrate, 0.05% Tween 20, pH 6.0) at 95°C for 30 min for antigen retrieval. Then, sections were washed with distilled water, and permeabilized with 0.1% Triton X-100 in PBS at room temperature for 45 min. Sections were blocked in 5% goat serum in PBS for 1 h at room temperature and incubated for overnight at 4°C with primary antibody solution as described in Table 2. The following day, tissue sections were washed three times with PBS and incubated with secondary antibody solution (Table 2) for 1 h at room temperature. Sections were then washed three times with PBS and counter-stained with TO-PRO-3 iodide at room temperature for 15 min (1:1000 dilution). Finally, tissue samples were analyzed using a confocal Zeiss LSM 700 microscope using a 40× objective.

**Table 2:** List of antibodies.

Antibody	Dilutions
Rabbit anti-aquaporin 5	200
Mouse anti-cytokeratin 7	500
Rabbit anti-TMEM-16A	100
Mouse anti-Na^+^/K^+^-ATPase α antibody	200
Rabbit anti-PECAM-1	100
Mouse anti-β-tubulin III	100
rabbit anti-Ki67	200
Alexa Fluor 488 conjugated anti-rabbit IgG	500
Alexa Fluor 568 conjugated anti-mouse IgG	500

Antibodies used for antigen detection in the immunohistochemistry.

### Proliferation assay

Confocal images of Ki67 stained tissue samples were captured at 40× magnification using a confocal Zeiss LSM 700. The number of Ki67 positive cells was counted using ImageJ software. Statistical significance was assessed by one-way ANOVA (*p* < 0.01) and Dunnett's post-hoc test for multiple comparisons to the sham control group.

## Results

### Scaffold mechanical property and stability

**Fig 2:**
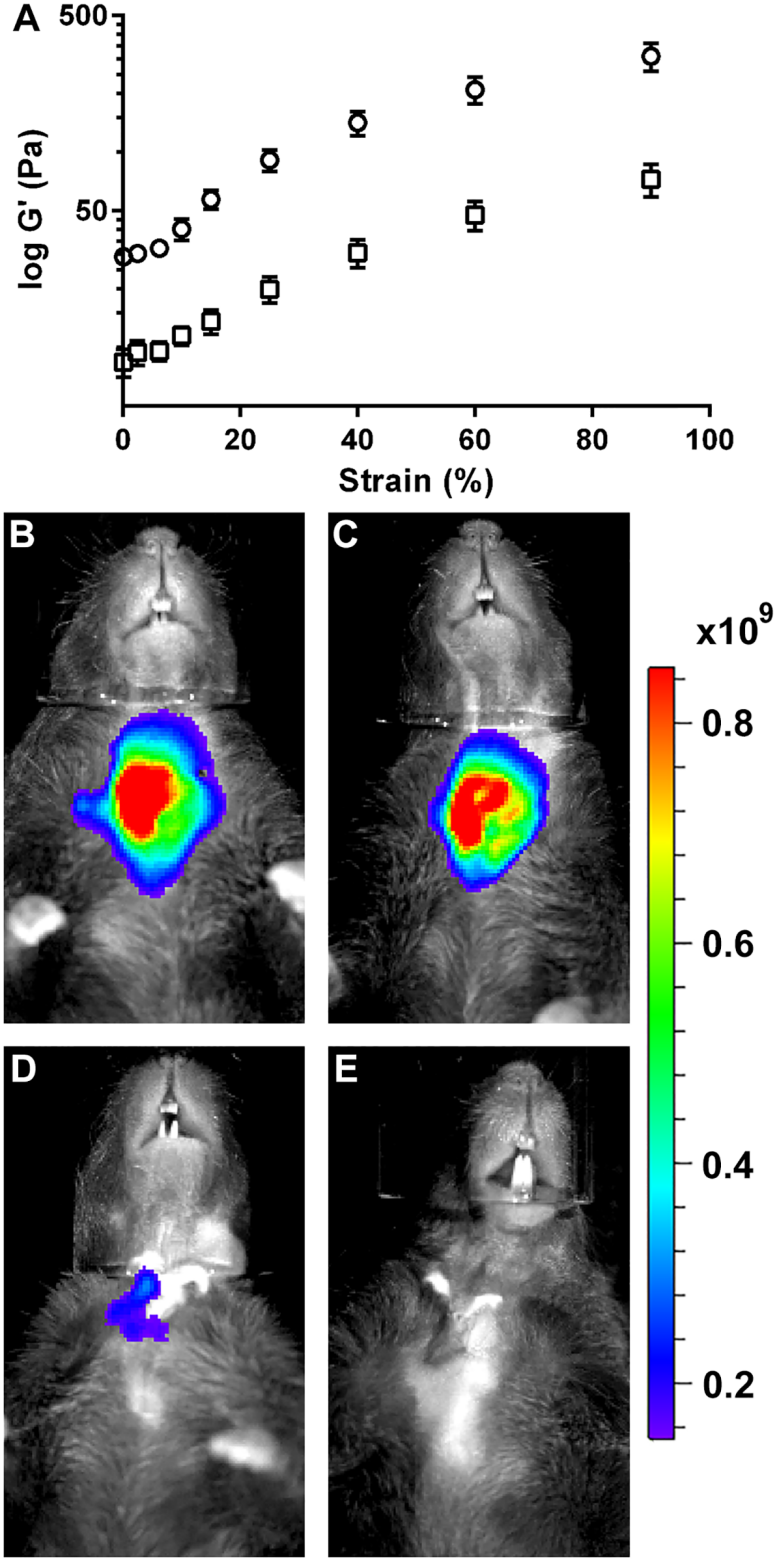
L_1p_-FH successfully attach to mSMG and are degraded over time *in vivo*. (A) Rheology measurements were performed for FH alone as well as L_1p_-FH. Data represent the elasticity (G’) versus strain (%) of unmodified FH (○) and L_1p_-FH (□). The *in vivo* stability of L_1p_-FH was monitored using a Xenogen IVIS 100 Bioluminescent Imager at days (B) 1, (C) 3, (D) 8 and (E) 20. Radiant Efficiency: (p/sec/cm^2^/sr)/(μW/cm^2^).

Rheology measurements showed that L_1p_-FH display a significant decrease in elasticity as compared to unconjugated FH thereby making a softer structure (Fig 2A). Moreover, the fluorescent intensity of FH at day 3 (Fig 2C) was similar with the post-surgery day 1 group (Fig 2B). However, the fluorescent intensity of the FH at day 8 (Fig 2D) was approximately 6 times lower as compared to post-surgery day 1 or day 3 groups (Fig 2B and 2C). Moreover, the fluorescent intensity of FH at day 20 (Fig 2E) was undetectable. These results suggest successful attachment of FH scaffold in the wounded tissue (*i*.*e*., high stability) and likely degradation over time *in vivo*.

### Measurement of body weight

**Fig 3:**
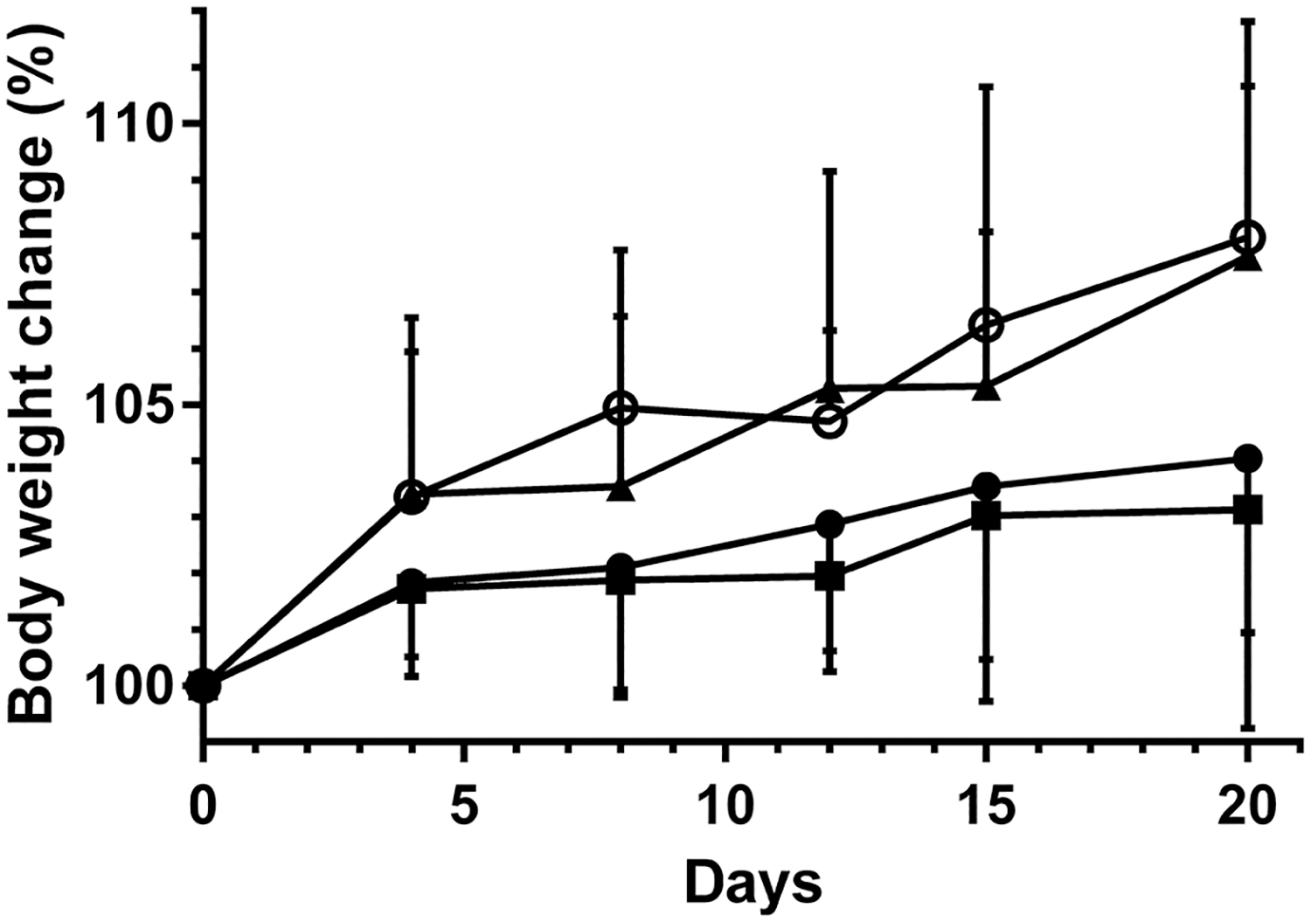
L_1p_-FH applied to mSMG increased body weight. Changes in body weight (%) of FH alone (■) or L_1p_-FH (▲) treated mice groups were compared with untreated mice group (●) and sham control group (○) over 20-day period. Data represent the means ± SD of n = 7 mice per condition and statistical significance was assessed by two-way ANOVA (*p* < 0.01) and Dunnett's post-hoc test for multiple comparisons to the untreated group.

Since saliva is important in eating, swallowing, and digestion, we sought to determine the ability of the mice to eat by measuring body weight at different times after surgery. We found no significant weight difference between untreated mice and FH alone treated mice (Fig 3). However, mice treated with L_1p_-FH had similar weights as the sham control group, which were significantly higher as compared to untreated mice and FH alone treated mice (*p* < 0.01).

### Saliva flow rate

**Fig 4:**
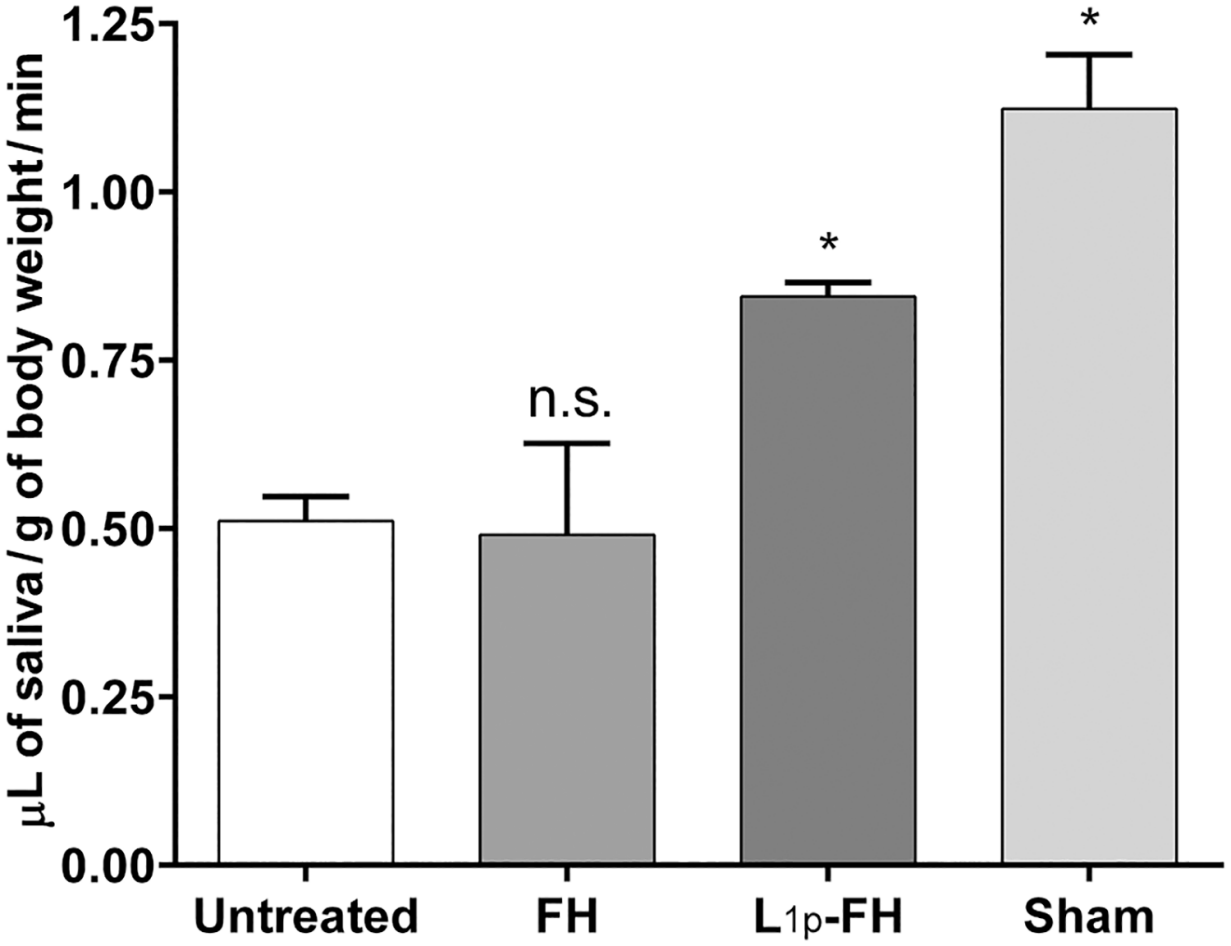
L_1p_-FH applied to mSMG improved saliva secretion over untreated and FH alone-treated mice. Mice were anesthetized and stimulated with pilocarpine at day 20. Then, saliva was collected for 5 min. Data represent the means ± SD of n = 5 mice per condition and statistical significance was assessed by one-way ANOVA (*p* < 0.01) and Dunnett's post-hoc test for multiple comparisons to the untreated group. * = significant difference from the untreated group; n.s. = no significant difference from the untreated group.

As shown in Fig 4, animals with no scaffold (untreated) or with FH alone displayed a significant decrease in saliva secretion rates (44% vs sham). In contrast, mice treated with L_1p_-FH showed a significant increase in saliva secretion rates as compared to untreated and FH alone-treated mice. Moreover, L_1p_-FH-treated mice showed increased saliva flow rates (75%) to levels close to sham controls (open incision but no surgical wound).

### Saliva protein composition

**Fig 5:**
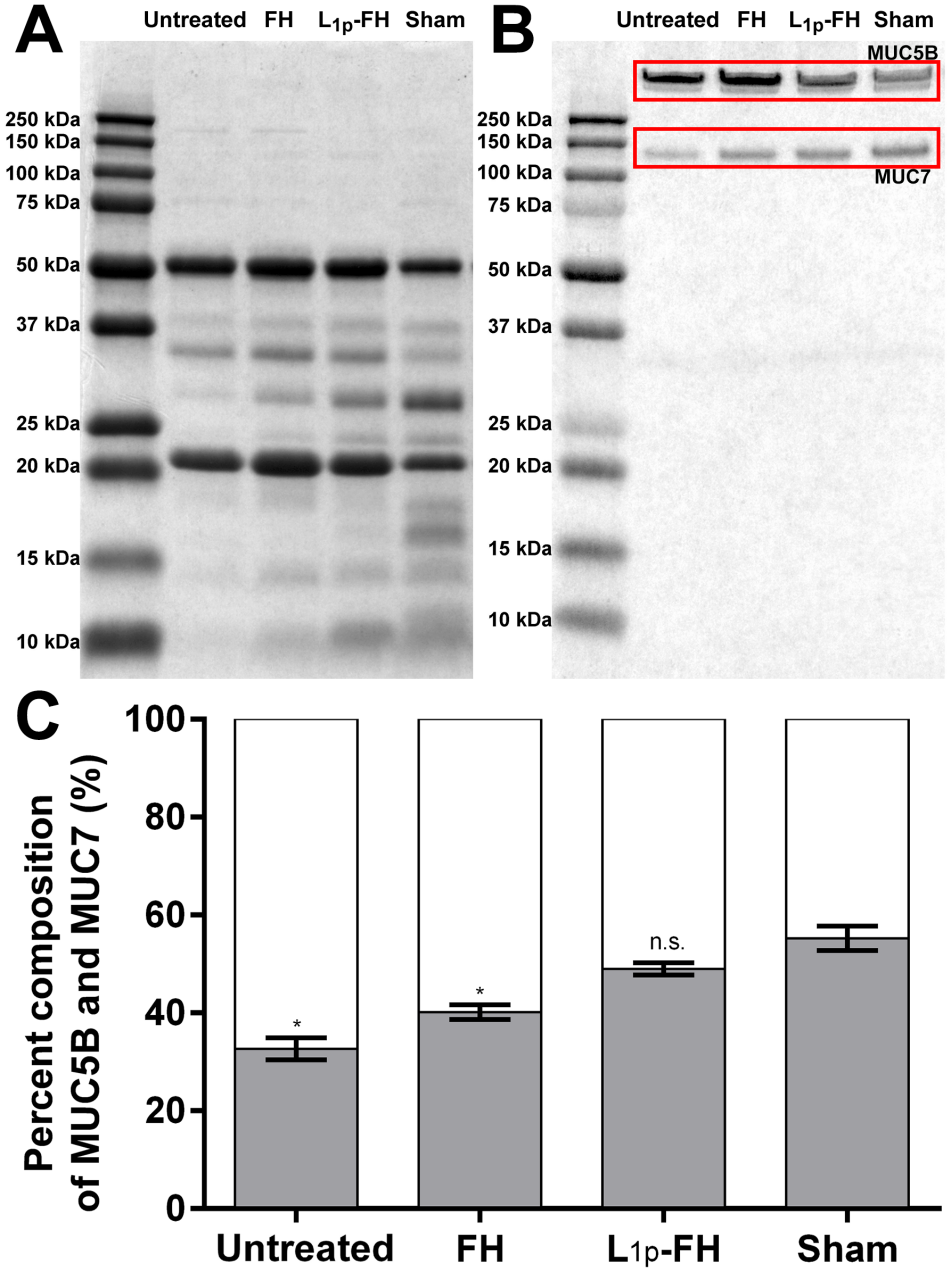
L_1p_-FH applied to mSMG restored saliva composition. Fifteen microgram of saliva protein from each group was fractionated by SDS-PAGE. The gel was stained with (A) 0.25% Coomassie Brilliant Blue R-250 for total proteins and (B) 0.5% Alcian Blue 8GX for mucins. (C) The mucin compositions were analysed using ImageJ. The white bar indicates MUC5B and the gray bar indicates MUC7. Statistical significance was assessed by one-way ANOVA (*p* < 0.01) and Dunnett's post-hoc test for multiple comparisons to the sham group. * = significant difference from the sham group; n.s. = no significant difference from the sham group.

We analyzed the protein composition in the stimulated saliva using SDS-PAGE. The total protein (Fig 5A) and mucin (Fig 5B) composition of the saliva from untreated and FH alone group showed clearly different patterns compared to the saliva from sham control group. The untreated group displayed decreased proline rich protein (15 kDa ~ 30 kDa) and cystatin (10 kDa) levels. In addition, animals with no scaffold or with FH alone displayed a significant decrease in MUC7 (*p* < 0.0001). However, the protein patterns of the L_1p_-FH treated group showed comparable protein patterns to sham control (Fig 5C). Moreover, the ratio of MUC5B and MUC7 in the saliva slightly differed between sham and L_1p_-FH treated group (*p* = 0.0111). These results indicate that the L_1p_-FH treated SMG could produce a similar quality of saliva as compared to sham controls.

### Histopathological studies

**Fig 6:**
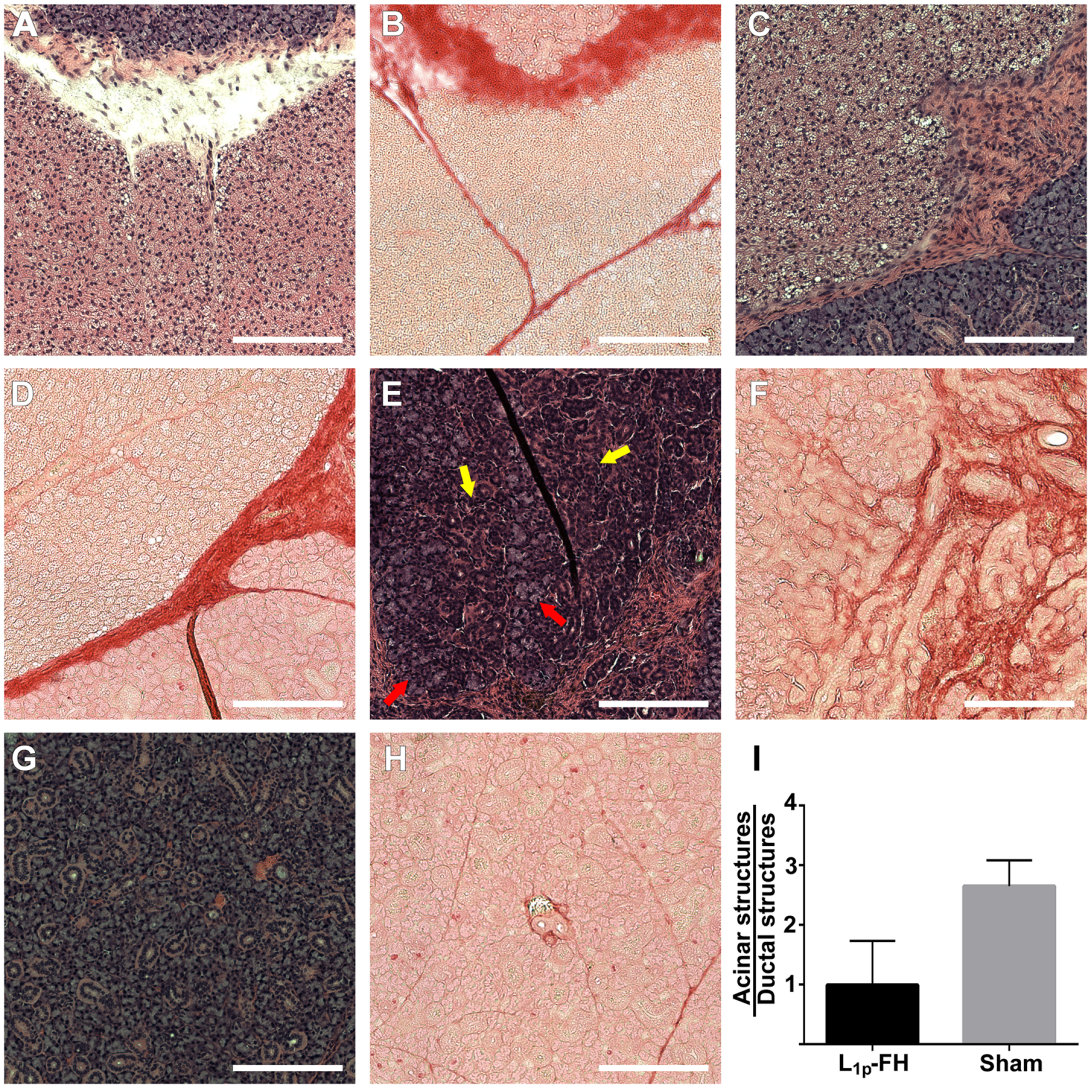
Surgical wounds treated with L_1p_-FH displayed organized mSMG. Rehydrated sections were stained with hematoxylin-eosin (A, C, E, G) or picrosirius red (B, D, F, H) stains and analyzed using a Leica DMI6000B at 10× magnifications. Shown are wounded mSMG without scaffold (A, B), wounded mSMG with FH alone (C, D), wounded mSMG with L_1p_-FH (E, F), and sham control (G, H). (I) The ratio of acinar and ductal structures was analyzed using ImageJ. Red arrows indicate acinar structures and yellow arrows indicate ductal structures. Scale bars = 200 μm.

To determine whether L_1p_-FH promoted tissue regeneration of mSMG surgical wounds *in vivo*, mSMG tissue sections were stained with H&E and picrosirius red. As shown in Fig 6, mSMG surgical wounds covered with L_1p_-FH displayed organized round acinar (red arrows) and ductal structures (yellow arrows) (Fig 6E) with organized collagen formation (Fig 6F). In contrast, wounded mSMG treated with no scaffold and FH alone formed disorganized collagen and failed to form organized round structures (Fig 6A–6D). A single blind histopathological analysis revealed that all mSMG treated with FH in general showed no differences in proliferation rates between the different groups and lack of evidence for cellular atypia. Additionally, the ratio of acinar and ductal structures is comparable to sham controls (Fig 6I).

### Confocal microscopy

**Fig 7:**
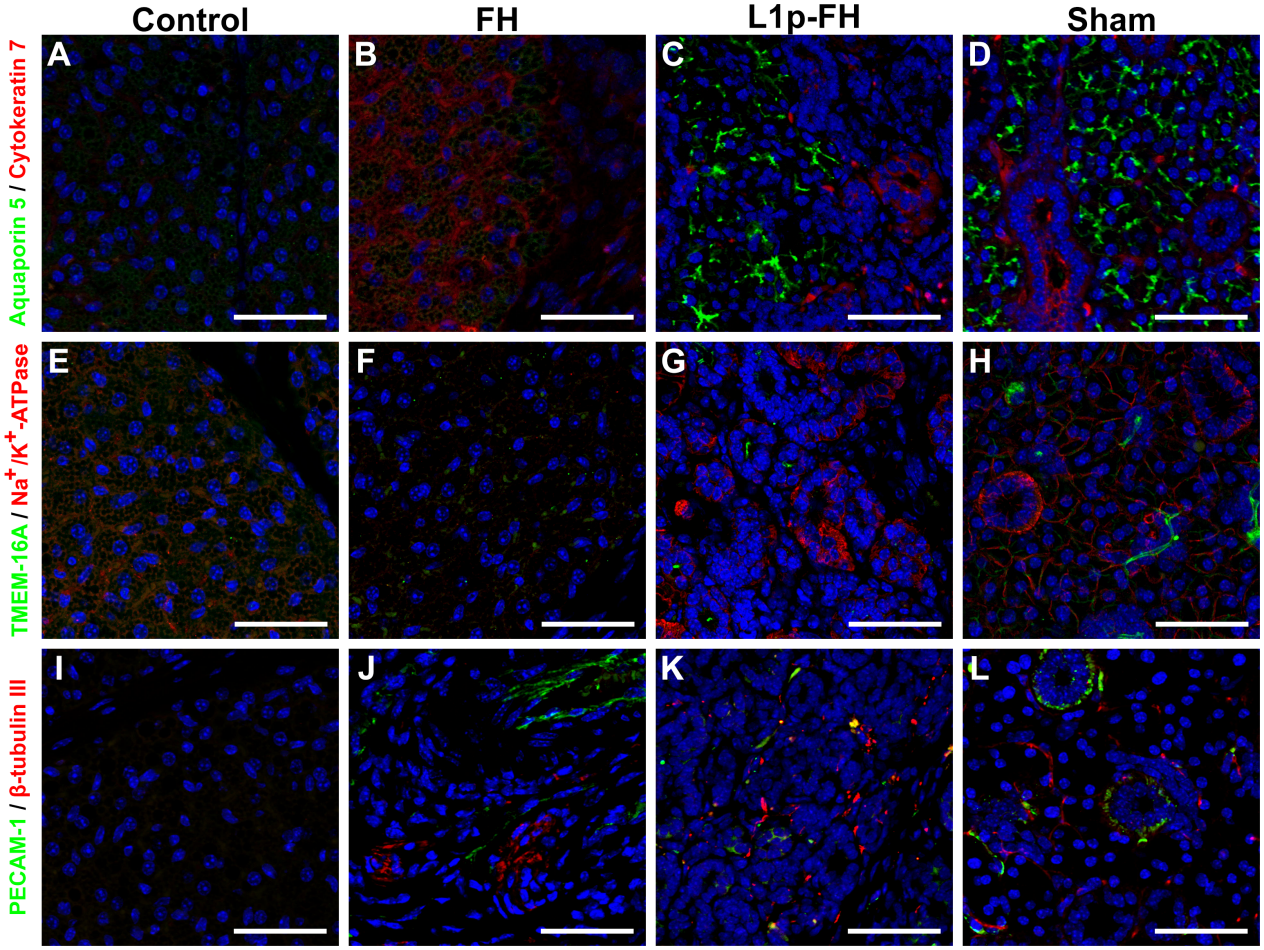
Acinar and ductal markers were expressed in the regenerating mSMG. Salivary structural and functional marker organization in wounded mSMG without scaffold (A, E, I), wounded mSMG with FH alone (B, F, J), wounded mSMG with L_1p_-FH (C, G, K), and sham control (D, H, L) was determined using Confocal microscopy as follows: (A-D; green) rabbit anti-aquaporin 5 and (A-D; red) mouse anti-cytokeratin 7, (E-H; green) rabbit anti-TMEM-16A and (E-H; red), mouse anti-Na^+^/K^+^-ATPase, (I-L; green) rabbit anti-PECAM-1 and (I-L; red) mouse anti-β-tubulin III. Scale bars = 50 μm.

To verify whether mSMG surgical wounds covered with L_1p_-FH regenerated salivary epithelium, mSMG sections were stained with following markers: aquaporin 5 (water channel protein, acinar marker), cytokeratin 7 (ductal epithelial marker), TMEM16A (apical chloride transporter), Na^+^/K^+^-ATPase (basolateral membrane marker), PECAM-1 (endothelial cell marker) and β-Tubulin III (neuronal cell marker). As shown in Fig 7, the apical acinar cell marker (aquaporin 5, green) and the ductal cell marker (cytokeratin 7, red) were detected in the L_1p_-FH treated group (Fig 7C) and sham control group (Fig 7D). Conversely, untreated (Fig 7A) or FH alone treated (Fig 7B) wounds displayed very weak aquaporin 5 and disorganized cytokeratin 7 staining. Moreover, surgical wounds covered with L_1p_-FH showed apical TMEM16A (green) and basolateral Na^+^/K^+^-ATPase localization (red) (Fig 7G) but untreated (Fig 7E) or FH alone treated (Fig 7F) wounds displayed very weak expression or no staining at all. For endothelial and neuronal markers, untreated wounds displayed poor staining (Fig 7I) and FH alone treated wounds showed disorganized structure (Fig 7J). Interestingly, L1p-FH treated wounds showed endothelial marker signals (green) and some line structure of β-tubulin III (red) indicating the presence of small capillaries and neurons (Fig 7K).

### Proliferation assay

**Fig 8:**
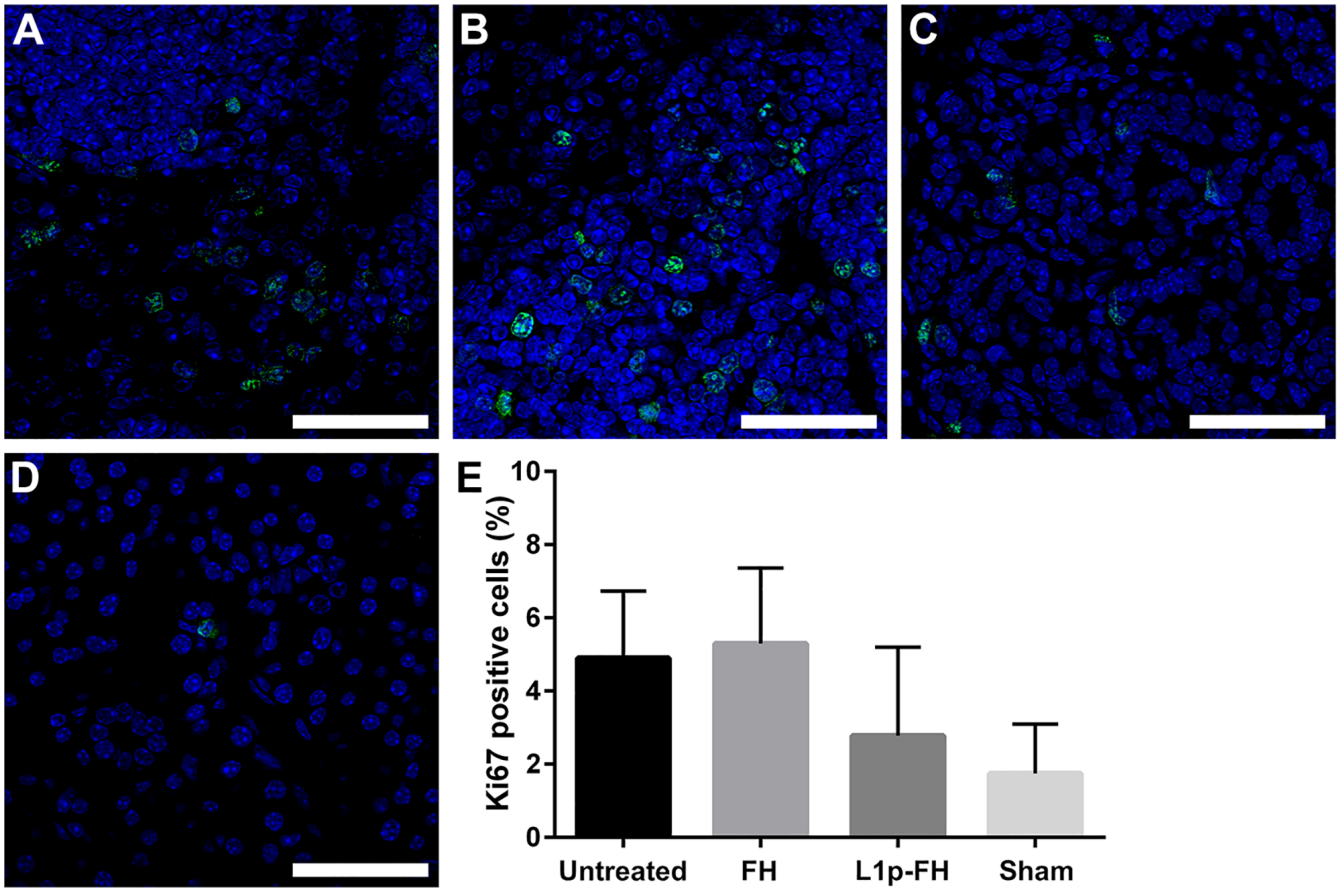
L_1p_-FH does not promote abnormal cell proliferation. The proliferation marker Ki67 showed similar staining patterns in wounded mSMG without scaffold (A), wounded mSMG with FH alone (B), wounded mSMG with L_1p_-FH (C), and sham control (D). Statistical significance was assessed by one-way ANOVA (*p* < 0.01) and Dunnett's post-hoc test for multiple comparisons to the sham group (E). Scale bars = 50 μm.

Our previous studies demonstrated that L_1p_-FH treated tissues displayed a significantly higher number of Ki67 positive cells as compared with untreated and FH treated controls on post-surgery day 8 [24]. However, L_1p_-FH, FH alone -treated tissues as well as no-scaffold controls showed no significant differences as compared to sham controls on post-surgery day 20 (Fig 8). Moreover, our statistical analyses showed no significant difference in cell proliferation between the different groups.

## Discussion

Recently, we published a study utilizing a mouse model for wounded mSMG to demonstrate that L1 peptide conjugated FH contributes to tissue regeneration [24]. In the previous study, regenerated tissue showed a strong expression of structural protein cell markers; however, the signals for functional cell markers were weak indicating incomplete wound healing. We believe this was due to a short monitoring time-frame. Therefore, we decided to test whether L_1p_-FH could restore both tissue structure organization and function when treatments lasted for longer periods. In fact, the monitoring of L_1p_-FH over time indicates that even though L_1p_-FH was significantly softer than FH (Fig 2A), it can still successfully attach to wounded SMG, is stable for several days and the gel is degraded over time *in vivo* (Fig 2B–2E). Moreover, our results indicated that L_1p_-FH are able to almost completely restore functional markers in the wounded mSMG as compared to previous studies [24]. While the mechanisms by which this process occurs are not fully understood, previous studies have shown that L1 peptides bind to α_3_β_1_, α_6_β_1_, α_6_β_4_ and α_7_β_1_ integrins, while fibrinogen binds to αIIbβ_3_ and α_5_β_1_ integrins [26–28]. These integrins are present in epithelial tissues and their activation induces cell migration, proliferation, and adhesion [29–32].

The main clinical concern in patients with salivary gland damage is the reduction of salivary flow [33]. Moreover, salivary gland defects are highly related to weight loss as saliva is necessary for eating, chewing, swallowing and digesting [34, 35]. Therefore, in this study we demonstrated that animals with untreated with untreated SMG wounds not only showed significant reduction of saliva flow (Fig 4) but also displayed significant weight loss (Fig 3), while treatment with L_1p_-FH completely abolished these problems. These results suggest that L_1p_-FH was effective in promoting salivary gland function. To prevent misinterpretation of the data shown above, we were able to use a sham control group that was comparable to healthy mice that showed no weight loss or signs of infection (data not shown).

Mucins are high molecular weight glycoproteins secreted by salivary glands. The best studied mucins in the human saliva are a) MUC5B (a larger salivary gland mucin) which is present in submandibular gland (SMG) secretions and is thought to be related to the perception of dry mouth (*i*.*e*., by retaining moisture in the mucosa) and b) MUC7 (a smaller salivary gland mucin) appears to have a role in preventing bacterial attachment to the enamel and mucosal surfaces [36]. Moreover, MUC7 is less abundant in the gel-phase, making saliva less viscous and therefore contributing to the natural saliva rheology [37]. MUC7 appears to be undetectable in saliva from minor glands; however, it is present in saliva from SMG and sublingual glands [38]. In this study, we found that animals with no scaffold or with FH alone displayed a significant increase of MUC5B protein levels and a significant decrease MUC7 protein levels as compared to the sham control group while treatment with L_1p_-FH restored MUC5B and MUC7 expression to levels comparable to sham controls (Fig 5). This result indicates that L1 peptides are able to restore saliva component and saliva rheology.

Collagen acts as a structural scaffold in epithelial tissues, also controls many cellular functions (*e*.*g*. cell migration, differentiation, and synthesis of proteins) [39, 40]. Therefore, collagen organization plays a critical role in the wound healing process [41, 42]. Our results showed that L_1p_-FH was able to promote organized cell structure and collagen formation (Fig 6). We speculate that these effects are due to L_1p_-FH’s ability to bind and attract stem/progenitor cells through interactions with integrins described above, and thus accelerating wound healing. However, when wound healing is incomplete or excessive it may can cause unwanted side effects. Specifically, it was reported that inflammation pathways during wound healing may promote growth and survival of cancer stem cells [43]. However, our results showed no differences in cell proliferation between the studied groups (Fig 8). Moreover, a single blinded histopathological study described an absence of cellular atypia. Together these results indicate that the regeneration process using L_1p_-FH is controlled and could be used safely in other species.

Regarding the expression of functional cell markers, we found a strong aquaporin 5 signal in the regenerated mSMG (Fig 7C). Since this protein transports water across the cell membrane during fluid secretion, its expression is also essential for SMG regeneration [44, 45]. In addition, TMEM16A (apical chloride transporter), Na^+^/K^+^-ATPase (basolateral antiport transporter) were expressed in the regenerated mSMG (Fig 7G). These proteins are responsible for maintaining the proper ionic composition for saliva secretion and are also critical for SMG functioning [46, 47]. Finally, we detected PECAM-1 and β-Tubulin III signal in the regenerating gland (Fig 7K), indicating formation of blood and nerves, respectively, which have been shown to regulate SMG development [48]. Our results indicate that L_1p_-FH are able to promote SMG regeneration *in vivo* and are consistent with previous studies [21, 24, 49]; however, here we found better regeneration endpoints due to longer treatments times. These studies are highly significant as they could offer viable path forward for advancing the treatment of hyposalivation.

## Conclusion

In summary, we demonstrated that FH modified with L1 peptides facilitated salivary gland tissue healing. Our results suggest that L_1p_-FH is suitable for *in vivo* applications (as it is both biodegradable and biocompatible and significantly accelerates formation of new tissue as compared to FH alone or no scaffold). The regenerated gland tissues displayed not only structural but also functional similarities to normal gland tissues. As stated, the increased duration of the current experiment allowed for additional developments to occur and for more definitive data to emerge. The current results suggest that L1p-FH may have activated multiple cellular processes that contributed to tissue regeneration.
